# Supplementary material for: Non-biological synthetic spike-in controls and the AMPtk software pipeline improve mycobiome data
Source: PeerJ. 2018 May 28;6:e4925. doi: 10.7717/peerj.4925 (PMC5978393; doi:10.7717/peerj.4925)
Supplement: Supplemental Information 1 [file peerj-06-4925-s001.docx]

## Supporting Information

Article title: Non-biological synthetic spike-in controls and the AMPtk software pipeline improve mycobiome data

Authors: Jonathan M. Palmer, Michelle A. Jusino, Mark T. Banik, and Daniel L. Lindner

The following Supporting Information is available for this article:

**Fig. S1** Read abundances do not correlate with actual abundances**.**

**Fig. S2** Random subsampling reads for each sample does not improve accuracy of read abundances.

**Fig. S3** Index-bleed on Illumina MiSeq occurs during the sequencing run and is not a result of contamination.

**Table S1** Cultures from the CFMR culture collection used to construct the BioMock community.

**Table S2** OTU clustering results using default QIIME pre-processing of reads.

**Table S3** Expected errors quality trimming removes most errors from Ion Torrent PGM data using 12 member SynMock community.

**Table S4** Expected errors quality trimming removes most errors from Illumina MiSeq data using 12 member SynMock community.

**Table S5** Summary statistics for the HTAS runs used in this study.

**Fig. S1.** Read abundances do not correlate with actual abundances even when DNA concentration is high and PCR cycles are low. Creating libraries of the equimolar BioMock community by varying PCR cycles and DNA concentrations for sequencing on the Ion Torrent PGM did little to change read abundances. However, these data are consistent with traditional recommendations to use as few PCR cycles as possible during library prep.

**Fig. S2.** Random subsampling reads for each sample does not improve accuracy of read abundances. Each sample was randomly sub-sampled to 100,000 reads using ‘amptk sample’ and then reads were mapped to the BioMock community. Chi-square test for each of these BioMock samples was significant (p < 0.001), indicating the read abundances are not equally distributed.

**Fig. S3.** Index-bleed on Illumina MiSeq occurs during the sequencing run and is not a result of contamination. Sequencing the BioMock on Illumina MiSeq resulted in elevated levels of apparent index-bleed during our first run. To rule out that this was a result of contamination during library prep/cleanup, the same libraries were sequenced on a second run in the absence of any of the BioMock samples. The index-bleed discovered in the first run then disappeared, however, one of the BioMock members (mock3) was actually found in these environmental samples.

**Table S1** Cultures from the CFMR culture collection used to construct the BioMock community.

| Species | Voucher ID | Mock ID | ITS2 Length | % GC | GenBank Accession |
| --- | --- | --- | --- | --- | --- |
| *Phialocephala fusca* | FP-170182 | mock1 | 237 | 68.35% | KU668953 |
| *Ascomycete sp.* | FP-170235 | mock2 | 238 | 50.84% | KU668968 |
| *Phialocephala lagerbergii* | FP-170134 | mock3 | 238 | 58.82% | KU668951 |
| *Helotiales sp* | RF10JR | mock4 | 239 | 57.32% | KU668958 |
| *Aspergillus candidus* | RF1JR | mock5 | 260 | 65.77% | KU668969 |
| *Bjerkandera adusta* | RF3JR | mock6 | 281 | 51.25% | KU668970 |
| *Laetiporus caribensis* | GDL-1 | mock7 | 283 | 52.65% | KU668960 |
| *Trametes gibbosa* | RF5JR | mock8 | 288 | 50.00% | KU668971 |
| *Laetiporus gilbertsonii* | OR-2 | mock9 | 290 | 54.14% | KU668967 |
| *Gloeporus pannocinctus* | MR5-1 | mock10 | 292 | 43.84% | KU668965 |
| *Wolfiporia dilatohypha* | FP-72162 | mock11 | 293 | 54.61% | KU668959 |
| *Schizopora sp.* | FP-170198 | mock12 | 293 | 48.12% | KU668955 |
| *Fomitopsis ochracea* | FP-170231 | mock13 | 295 | 44.07% | KU668957 |
| *Laetiporus cermeioporus* | L34-2 | mock14 | 296 | 54.73% | KU668963 |
| *Phanerochaete laevis* | RF9JR | mock15 | 300 | 47.67% | KU668973 |
| *Laetiporus cincinnatus* | DA-37 | mock16 | 302 | 53.97% | KU668950 |
| *Punctularia strigosozonata* | RF7JR | mock17 | 303 | 53.14% | KU668972 |
| *Phellinus cinereus* | IN4-1 | mock18 | 314 | 49.68% | KU668962 |
| *Antrodiella semisupina* | MR-3 | mock19 | 315 | 43.81% | KU668966 |
| *Leptoporus mollis* | TJV-93-174 | mock20 | 315 | 45.40% | KU668975 |
| *Leptoporus mollis 2* | RLG-7163 | mock21 | 315 | 45.08% | KU668974 |
| *Mortierellales sp* | FP-170186 | mock22 | 353 | 45.04% | KU668954 |
| *Laetiporus persicinus* | HHB-9564 | mock23 | 379 | 51.19% | KU668961 |
| *Penicillium nothofagi* | FP-170215 | mock24 | 260 | 66.15% | KU668956 |
| *Metapochonia suchlasporia* | FP-170177 | mock25 | 291 | 64.60% | KU668952 |
| *Wolfiporia cocos* | MD-275 | mock26 | 548 | 59.67% | KU668964 |

**Table S2** OTU clustering results using default QIIME pre-processing of reads.

| Platform | Clustering method | Reads | Total OTUs | Mock OTUs  (n = 12) | Error Rate (mismatches / total) |
| --- | --- | --- | --- | --- | --- |
| Ion Torrent PGM (400 bp) | UCLUST | 2 562 316 | 97 175 | 1 347 | 3.760% |
|  | USEARCH | 2 562 316 | 9 812 | 560 | 4.237% |
|  | SWARM | 2 562 316 | 276 403 | 225 | 3.517% |
|  | UPARSE | 2 562 316 | 1 609 | 82 | 1.100% |
|  |  |  |  |  |  |
| Illumina Miseq  (2 x 300) | UCLUST | 15 696 636 | 122 802 | 528 | 0.131% |
|  | USEARCH | 15 696 636 | 9 785 | 545 | 4.694% |
|  | SWARM | 15 696 636 | 614 133 | 165 | 4.447% |
|  | UPARSE | 15 696 636 | 2 483 | 38 | 0.077% |

**Table S3** Expected errors quality trimming removes most errors from Ion Torrent PGM data using 12 member SynMock community.^1^

| Method | Aligned reads | Subst. errors | Indel errors | UPARSE OTUs | OTUs  (chimera filtered) |
| --- | --- | --- | --- | --- | --- |
| No Qual Filter | 67 185 | 0.237% | 0.342% | 26 | 21 |
| Cutadapt -q 25 | 73 092 | 0.152% | 0.222% | 28 | 26 |
| Seqtk (Phred) | 75 535 | 0.204% | 0.314% | 83 | 79 |
| Sickle –q 25 | 71 221 | 0.098% | 0.087% | 31 | 30 |
| Exp. Errors < 1 | 35 810 | 0.078% | 0.100% | 18 | 14 |

^1^ Total of 78,525 reads from the SynMock Ion Torrent PGM run demuxed with AMPtk v1.0.1.

**Table S4** Expected errors quality trimming removes most errors from Illumina MiSeq data using 12 member SynMock community. ^1^

| Method | Aligned reads | Subst. errors | Indel errors | UPARSE OTUs | OTUs  (chimera filtered) |
| --- | --- | --- | --- | --- | --- |
| No Qual Filter | 1 081 931 | 0.333% | 0.006% | 44 | 27 |
| Cutadapt -q 25 | 1 148 274 | 0.253% | 0.007% | 361 | 337 |
| Seqtk (Phred) | 1 115 657 | 0.316% | 0.007% | 173 | 150 |
| Sickle -q 25 | 1 153 190 | 0.166% | 0.006% | 304 | 285 |
| Exp. Errors < 1 | 961 458 | 0.094% | 0.006% | 45 | 27 |

^1^ Total of 1,167,662 reads from the SynMock Illumina MiSeq run demuxed with AMPtk v1.0.1.

**Table S5** Summary statistics for the HTAS runs used in this study.

| Run | Platform | Total Reads | Valid Reads | Num Samples | Range reads per sample | Total UPARSE OTUs | Mock Community | Mock Calculated Error Rate | Index-Bleed |
| --- | --- | --- | --- | --- | --- | --- | --- | --- | --- |
| Mock3 | Ion Torrent PGM | 4,332,502 | 3,029,824 | 19 | 107,416 - 217,372 | 1,010 | BioMock | 0.086% | 0.033% |
| Mock4a | Illumina Miseq | 5,668,955 | 5,661,700 | 20 | 237,035 - 334,455 | 1,778 | BioMock | 0.019% | 0.264% |
| Mock4b | Illumina Miseq | 659,738 | 658,730 | 4 | 145,405 - 191,095 | 477 | None | NA | NA |
| Mock4c | Illumina Miseq | 6,103,680 | 6,096,296 | 20 | 221,130 - 392,118 | 1,625 | BioMock | 0.020% | 0.233% |
| Mock5 | Ion Torrent PGM | 4,341,392 | 2,602,544 | 21 | 59,394 - 254,269 | 927 | SynMock | 0.099% | 0.156% |
| Mock6 | Illumina Miseq | 18,058,078 | 17,911,411 | 21 | 621,228 - 1,163,433 | 2,470 | SynMock | 0.063% | 0.057% |
